# Enhanced photocatalytic performance of TiO_2_-ZnO hybrid nanostructures

**DOI:** 10.1038/srep04181

**Published:** 2014-02-25

**Authors:** Chun Cheng, Abbas Amini, Chao Zhu, Zuli Xu, Haisheng Song, Ning Wang

**Affiliations:** 1 Department of Materials Science and Engineering, South University of Science and Technology, Shenzhen, 518055, China; 2 Institute for Frontier Materials, Deakin University, Waurn Ponds, VIC 3217, Australia; 3 Department of Physics, Hong Kong University of Science and Technology, Hong Kong, China; 4 Wuhan National Laboratory for Optoelectronics (WNLO) and the School of Optical and Electronic Information, Huazhong University of Science and Technology, Wuhan 430074, China; 5 These authors contributed equally to this work.

## Abstract

We studied the photocatalytic properties of rational designed TiO_2_-ZnO hybrid nanostructures, which were fabricated by the site-specific deposition of amorphous TiO_2_ on the tips of ZnO nanorods. Compared with the pure components of ZnO nanorods and amorphous TiO_2_ nanoparticles, these TiO_2_-ZnO hybrid nanostructures demonstrated a higher catalytic activity. The strong green emission quenching observed from photoluminescence of TiO_2_-ZnO hybrid nanostructures implied an enhanced charge transfer/separation process resulting from the novel type II heterostructures with fine interfaces. The catalytic performance of annealing products with different TiO_2_ phase varied with the annealing temperatures. This is attributed to the combinational changes in E_g_ of the TiO_2_ phase, the specific surface area and the quantity of surface hydroxyl groups.

Semiconductor nanostructured composites draw intensive interests because of their improved properties and promising technological applications, such as photochemical solar cells, photo catalysts, photosensors, electro-luminescent displays, and biolabels. In recent years, considerable effort has been exerted toward combining semiconductor nanostructures with suitable materials to synergize their properties. For example, the deposition of metals or metallic islands on ZnO and TiO_2_ nanostructures can result in an enhanced photocatalytic performance^1^,^2^,^3^,^4^. Generally, photocatalytic efficiency is limited by a fast recombination of photogenerated charge carriers. In semiconductor/metal nanocomposites, the metal component can trap the photo-induced charge carriers and consequently promotes the interfacial charge-transfer processes^1^. Compared to the single element of ZnO and TiO_2_, coupled ZnO/TiO_2_ composite displays a largely improved photocatalytic activity, such as in the degradation of phenol, 2-chlorophenol, and pentachlorophenol^5^ and in the decomposition of salicylic acid^6^. This enhanced photocatalytic behavior is attributed to the efficient spatial separation of electrons and holes^7^. However, previous research works mainly studied the coupled ZnO/TiO_2_ with a simple core-shell structure (coated surface) or sandwich geometry (randomly adhered particles). Inappropriate structures of the interface in these two structures limit the interfacial charge-transfer process of photogenerated charge carriers and thus lower the photocatalytic property of the nanocomposite materials^8^,^9^,^10^,^11^. As such, challenges still remain in fabricating these nanohybrids with desirable interfacial structures. We have recently developed a simple method to build TiO_2_-ZnO (TZO) nanohybrid structures by a site-specific deposition of titanium oxide on ZnO nanorods (NRs)^12^. The TZO nanohybrids show uniform and atomically flat interfaces between ZnO and TiO_2_ with tunable crystal phases, which are amorphous, anatase and rutile. These TZO nanohybrid structures are expected to demonstrate an enhanced photocatalytic behavior. This is due to the improved interfacial structures which suit better the charge-transfer and spatial separation processes of photogenerated charge carriers. In this paper, we study the photocatalytic behaviors of TZO products and their annealed products with different TiO_2_ phase structures. The aim is to elucidate the relationship between various TZO structures and their photocatalytic properties.

## Results

**Figure 1:**
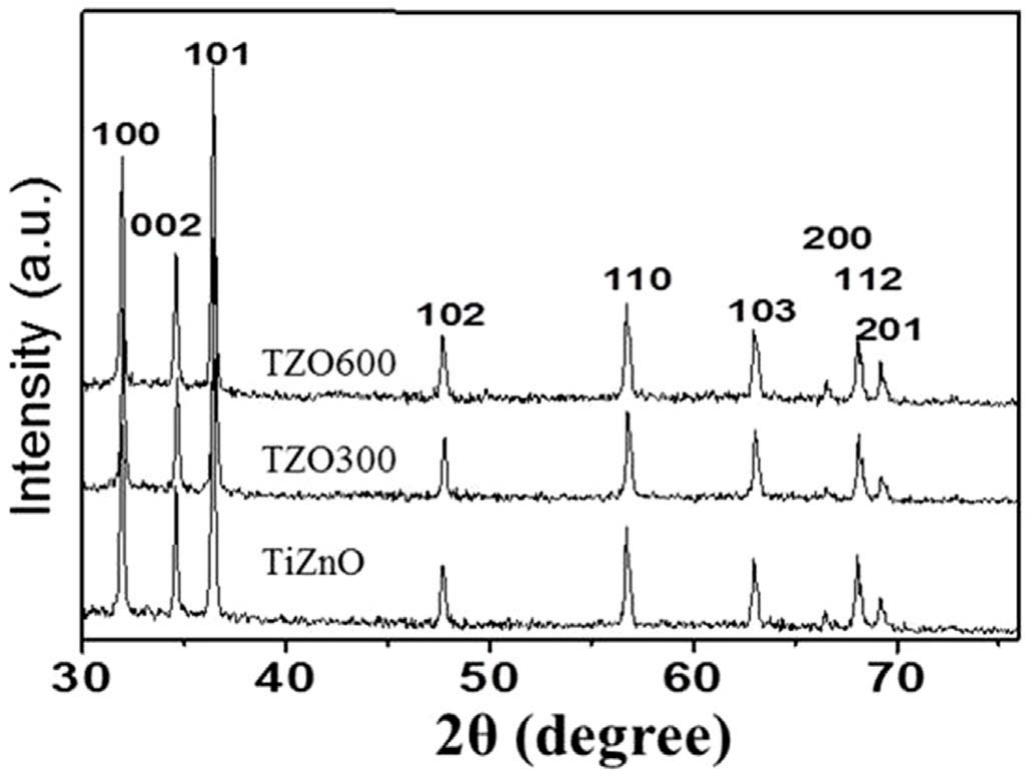
XRD patterns of TZO, TZO300 and TZO600. The prominent sharp peaks in all products are attributed to Wurtzite ZnO.

Figure 1 shows the XRD patterns of the produced TZO and the annealed TZOs, TZO300 and TZO600. The presence of Wurtzite-type hexagonal-phase ZnO is clearly shown in all samples with the lattice parameters a = 3.252Å and c = 5.208 Å. These data are in good agreement with the calculated values a = 3.253 and c = 5.209 (JCPDS 80-0075). There are no apparent peaks for anatase and rutile TiO_2_ in the XRD patterns of the annealed products. As the quantity of TiO_2_ coupled with ZnO NRs is very small, less than ~5 wt%, the XRD cannot detect them in the annealed substances.

**Figure 2:**
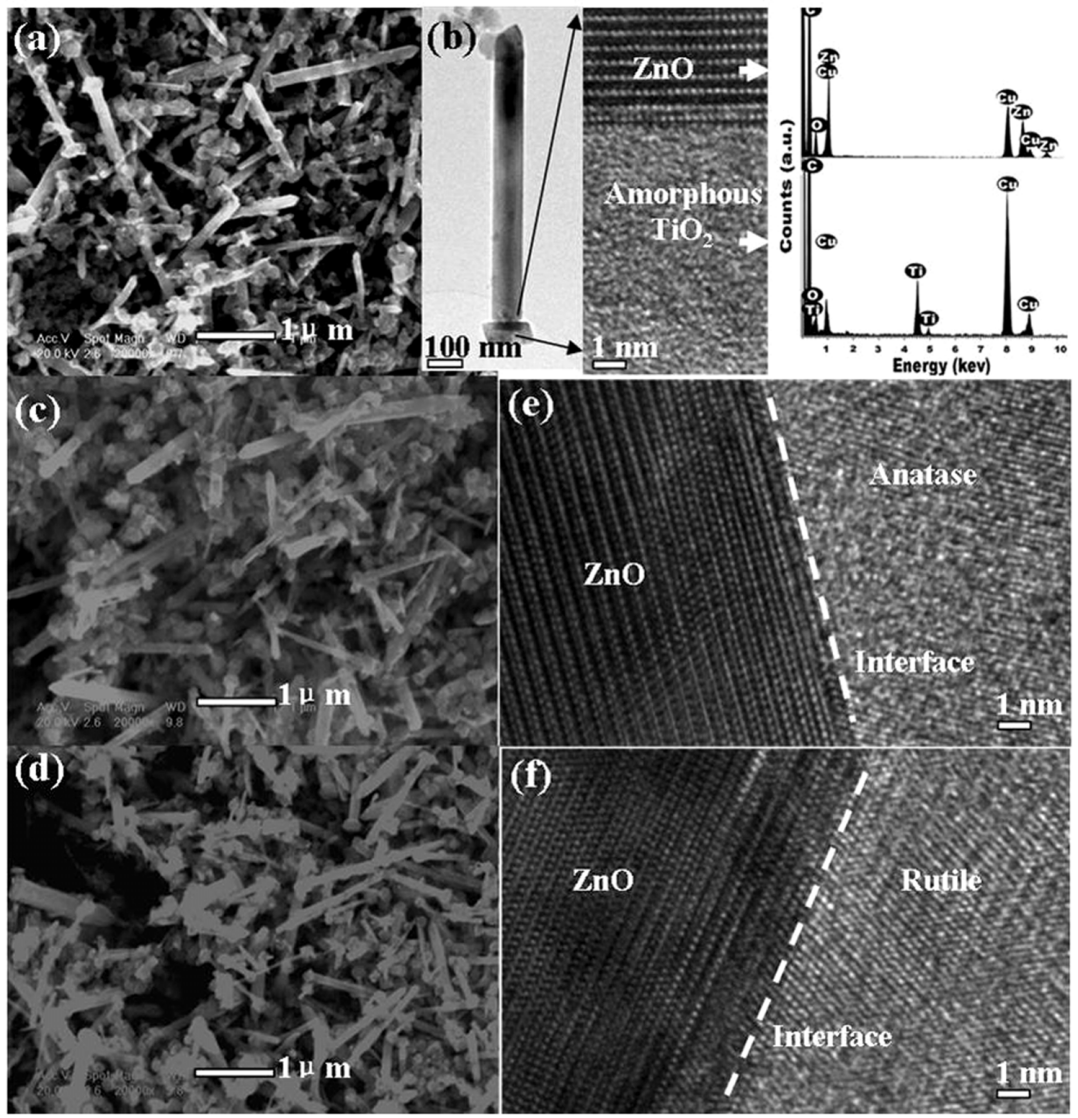
(a) SEM image of TZO, (b) TEM and HRTEM images of a TZO and its EDS spectra, (c) and (d) SEM images of TZO300 and TZO600, (e) and (f) HRTEM images of TZO300 and TZO600, displaying the fine structures of the interface.

The scanning electron microscopy images of TZO products in Fig. 2a reveal that TiO_2_ NPs with diameters in the range of 20–250 nm are deposited on one of the frontier ends of ZnO NRs. These ZnO NRs have diameters in the range of 10–200 nm and lengths in the range of 1–3 μm. Fig. 2 shows that the typical morphology of a single TZO nanohybrid containing a cap-like TiO_2_ NP which covers one end of the ZnO NR. The ZnO NRs are well crystallized with no detected impurities in the EDX results. The titanium oxide particles assembled with NRs are identified as amorphous, and the stoichiometric ratio of Ti to O is approximately 1:2 as measured by EDS (see Fig. 2b). The interfaces between ZnO NRs and TiO_2_ are flat and clearly visible when the electron beam is perpendicular to the NRs axes. After annealing, the products are still well dispersed and the morphologies do not change (Fig. 2c and d) while the amorphous TiO_2_ caps have transformed to the anatase and rutile phases. The interface structures of almost all of the nanohybrids are similar and atomically flat. Readers can refer to our previous work^12^,^13^ for more details about the formation mechanism of TZO and related structural characterizations.

**Figure 3:**
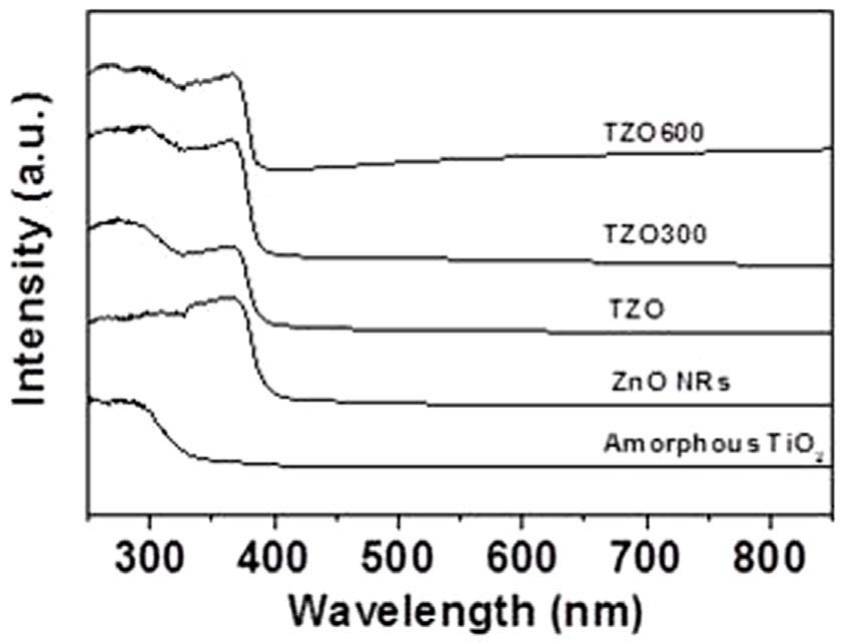
UV-Vis absorption spectra of amorphous TiO_2_ NPs, ZnO NRs, TZO nanohybrids and the annealed products TZO300 and TZO600.

The UV-Vis absorption spectra of amorphous TiO_2_ NPs, ZnO NRs, TZO nanohybrids and the annealed products at 300°C (TZO300) and 600°C (TZO600) are compared in Fig. 3. ZnO NRs and all TZOs have strong adsorption in the UV region at the absorption edge of ca. 380 nm, with a corresponding ZnO bandgap of 3.26 eV. The amorphous TiO_2_ NPs have a strong adsorption in the deep-UV region at the absorption edge ca. 313 nm with a corresponding bandgap of 3.96 eV^14^,^15^. For TZO, a higher absorbance below 320 nm ensures that the TiO_2_ NPs, which are attached to ZnO NRs, dominate the deep UV absorption. This may enhance the use of UV light compared with pure ZnO NRs.

**Figure 4:**
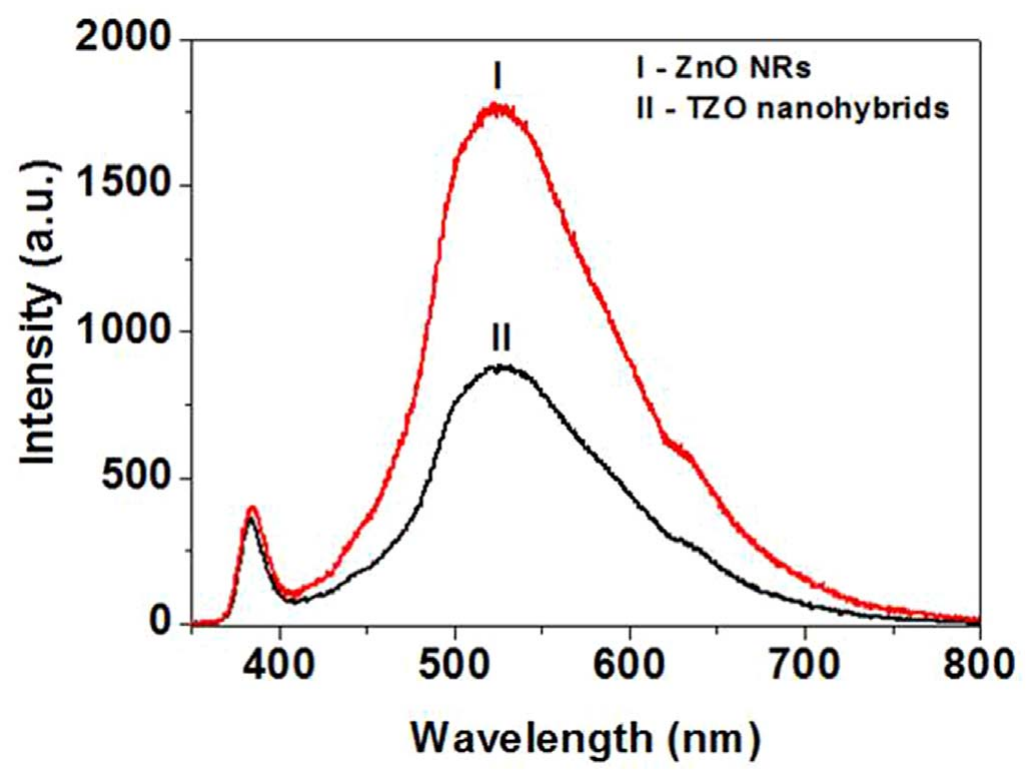
PL spectra of (I) ZnO NRs and (II) TZO nanohybrids.

Other studies on semiconductor-semiconductor and semiconductor-metal composites such as CdS-ZnO^16^, CdS-SWCNT^17^, ZnO-SWCNT^18^, CdS-AgI^19^, CdS-TiO_2_^20^, TiO_2_-Au^21^,^22^, and ZnO-Au^23^,^24^ have shown a charge equilibration when they are subjected to a bandgap excitation. Photo-induced electron transfer between composites can be initiated by the examined emission quenching (as shown in references 16,17,18,19,20,21,22,23,24). ZnO NRs with a bandgap of 3.26 eV undergo charge separation under UV-excitation (λ < 380 nm). These charge carriers (e_CB_ and h_VB_) can be either directly recombined or trapped in the vacancies (e_t_ and h_t_)^18^. Research has shown that the green emission from ZnO colloids originates from oxygen vacancies^25^. The green emission with a maximum of ~525 nm is a useful probe to monitor the charge transfer process on the ZnO surface. In the present study we monitor the green emission from ZnO to investigate the charge transfer interaction with TiO_2_. Fig. 4 shows the PL spectra of ZnO NRs and TZO nanohybrids. Due to the site-specific assembly of TiO_2_ NPs on ZnO NRs with fine interfaces, ZnO NRs are expected to intensely interact with the deposited TiO_2_, and as a result of this interaction we expect a decrease in the emission yield of ZnO. Indeed, due to this interaction, a significant decrease, ~ca. 50%, in the emission yield of the TZO nanohybrids is observed compared with that of pure ZnO NRs as shown in Fig. 4.

**Figure 5:**
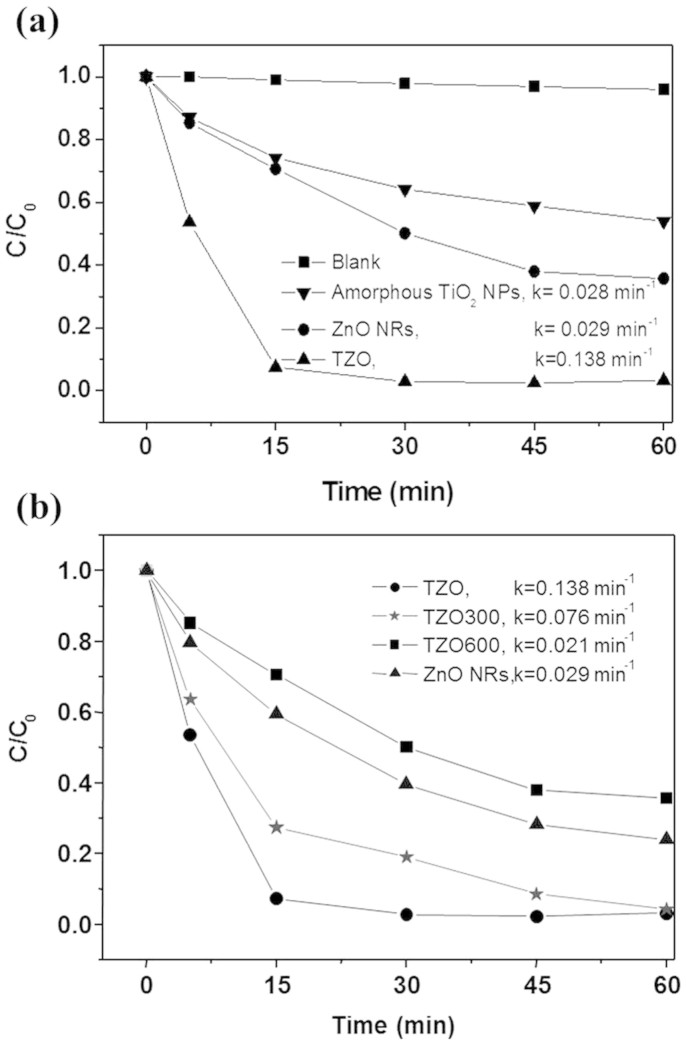
(a) Photodegradation of MB by amorphous TZO products, ZnO NRs, amorphous TiO_2_ NPs and blank experiment. (b) A comparison of the photocatalytic properties of ZnO NRs, TZO, TZO300 and TZO600.

Now, to evaluate the photo-oxidation capability of TZO nanohybrids, we examine the decomposition of MB dye in solution over the samples TZO, TZO300, and TZO600 under UV light irradiation as a function of time (Fig. 5). For comparison, we carried out the decomposition of the MB dye in a solution over two reference photocatalysts, amorphous TiO_2_ NPs and ZnO NRs under UV light irradiation (Fig. 5a). To show that the decomposition of MB dye over TZO is caused neither by catalysis nor photolysis, we carried out a decomposition experiment in a dark environment with TZO as well as under a full arc light irradiation (with no catalyst, blank experiment in Fig. 5a). In these experiments, the MB concentration remained unchanged as a function of time, that is, TZO is an active catalyst under UV light. From an exponential decay at the initial stage (0~15 min), the decomposition kinetics follows a first order kinetics with the classical equation ln(*C/C_0_*) = −*k***·***t*, where *k* is the so-called pseudo-first rate kinetic constant, *C_0_* is the initial concentration and *C* is the concentration after the MB degradation for time *t*. The presented *k* values in Fig. 5 are from fitting curves of the data for a 0–15 min period. These values represent a good measurement of the overall photo-degradation rate of all the investigated structures. TZO, with *k* = 0.138 min^−1^ which is about five times that of ZnO NRs, has the fastest MB decomposition rate. The photocatalytic activities of annealed products TZO300 and TZO600 decrease when the annealing temperature increases with the TiO_2_ phase on the TZO nanohybrid structures transformed from the amorphous to anatase and rutile phases.

## Discussion

**Figure 6:**
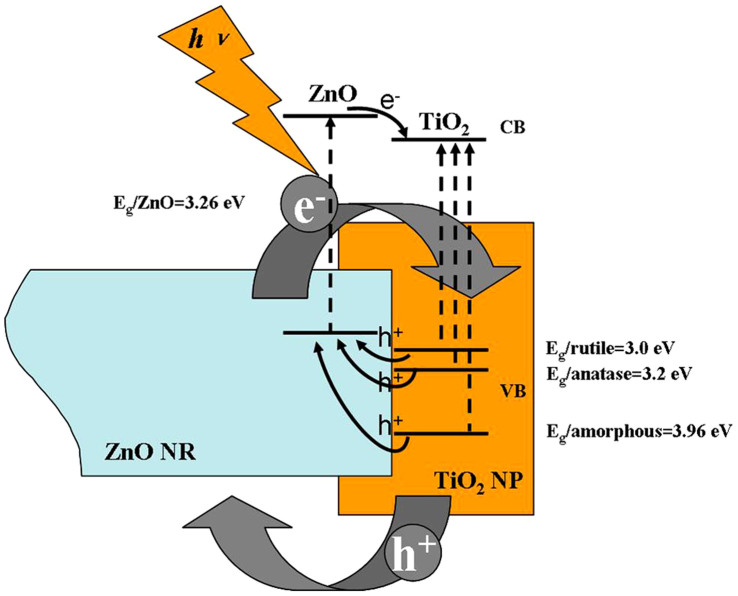
Illustration of photo-induced charge transfer and separation at the interface of TZO heterostructures.

The TZO nanohybrids form a type II semiconductor heterostructure^26^ as shown in Fig. 6. In this case, electrons and holes in semiconductors are at their lowest energy states. Therefore, the energy gradient at the interfaces tends to spatially separate those electrons and holes which are excited by UV light on different sides of the hetero-junction. Under illumination, the electrons are transferred from the conduction band (CB) of ZnO to CB of TiO_2_. In addition, the holes are transferred from the valence band (VB) of TiO_2_ to VB of ZnO. This process isolates active electrons and holes and, hence, accelerates the decrease in the electron-hole pair recombination and erodes the increase in lifespan. These phenomena directly result in an intense emission quenching as revealed by the PL results in Fig. 4. They also increase the availability of the pairs (electron and hole) on the surface of the photocatalysts and thus enhance the redox process. Significantly, the fine atomic flat interfaces across TiO_2_ and ZnO of TZO nanohybrids ease this charge transfer/isolation process by a decreased interface resistance. In addition, the elongated one dimensional structure of ZnO nanorods also helps to decrease the recombination probability of photogenerated carriers due to an increased delocalization of electrons^27^. Therefore, our special TZO structures favor the photocatalytic process not only by the band structure configurations but also by the fine interface structures and geometrical structures between the two components TiO_2_ and ZnO.

**Figure 7:**
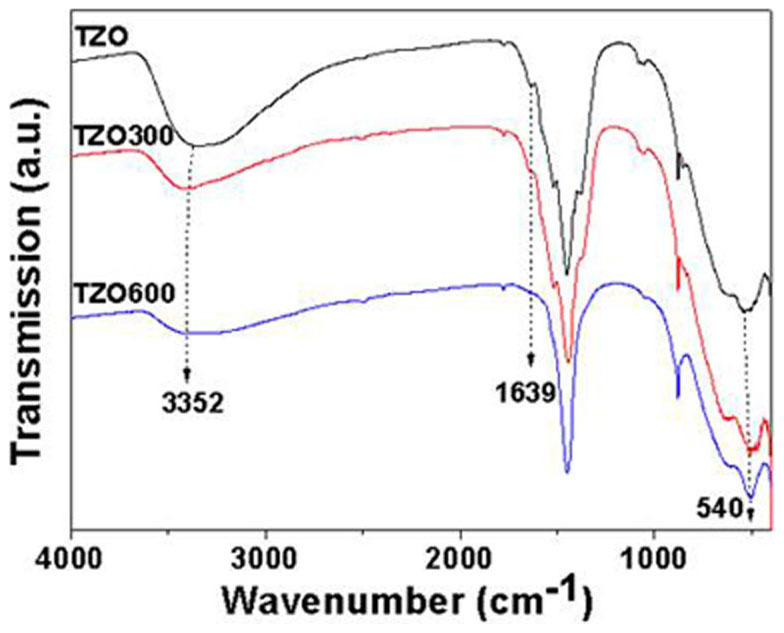
FTIR spectra of as-synthesized TZO, TZO300 and TZO600 samples in the wavenumber range of 4000–400 cm^−1^. The broad absorptions at about 3352 and 1639 cm^−1^ are assigned to the hydroxyl groups of chemisorbed and/or physisorbed H_2_O molecules on the samples. A strong absorption band near 540 cm^−1^ reveals the vibration properties of ZnO NRs. Other unsigned peaks are attributed to remnant organic species in the samples.

It is worthwhile to explain the rationale for the degeneration of the TZOs photocatalytic property sourced from the annealing treatment. First, as shown in Fig. 6, a larger energy gradient between VB_ZnO_ and VB_TiO2_ is apparently more beneficial for the transfer of active holes from TiO_2_ to ZnO. This difference is reduced as the bandgap energy (E_g_) of TiO_2_ structures drops after annealing treatments (E_g/rutile_ (3.0 eV) < E_g/anatase_ (3.2 eV) <E_g/amorphous_ (3.96 eV)). Hence, the ability of the charge transfer/separation is consequently weakened. Second, specific surface area and surface hydroxyl groups can strongly affect photocatalytic activities of nanomaterials and, sometimes, they are the dominating factors compared with the crystal structure^28^. In the present case, an increase in annealing temperature results in a decrease in the specific surface area of TZOs (TZO 14.643 m^2^/g; TZO300 13.763 m^2^/g; TZO600 8.418 m^2^/g). This temperature variation also decreases the quantity of the surface of hydroxyl groups (See caption of Fig. 7) which slows the photocatalytic activities. Nevertheless, the reaction rates of annealed TZOs are relatively higher than or close to that for ZnO NRs. These rationales determine the enhanced photocatalytic activities of TZOs.

In conclusion, this paper investigates the photocatalytic properties of TZOs and their annealed products with different TiO_2_ phases. Compared with the components of ZnO NRs and amorphous TiO_2_ NPs, the combined TZOs demonstrated higher catalytic activities. This is explained by an enhanced charge transfer/separation process resulting from the novel type II heterostructures with fine interfaces, and supported by the emission quenching mechanism in the PL studies. The catalytic performance of annealing products varied with the annealing temperatures. This originated from the combinational changes in E_g_ of the TiO_2_ phase, the specific surface area and the quantity of surface hydroxyl groups. Our investigation suggests an effective type of hybrid nanostructures for photocatalytic applications and other applications such as photodetectors and solar cells.

## Methods

The method for the fabrication of TZO nanohybrid structures is similar to that described in our recent work^12^ and consists of three steps: 1) Preparation of ZnO NRs: 5 mL of 0.1 M zinc acetate ethanol solution was mixed with 35 mL of 0.5 M NaOH ethanol solution to form a suspension solution. The suspension solution was later transferred to a 50 mL Teflon-lined stainless steel autoclave and kept at 180°C for 24 hours. 2) Preparation of sodium titanate nanotubes: 1 g of anatase TiO_2_ NPs were treated with a NaOH (10 M) aqueous solution in a Teflon vessel at 150°C for 12 hours^29^. 3) 200 mg sodium titanate nanotubes are mixed with the final reacted solution from step 1 and kept at 180°C for another 24 hours. Off-white precipitation products, obtained at the bottom of the autoclave, were sonicated for 30 minutes and then allowed to stand for another 30 minutes in order to separate them into two layers. The upper white TZO and the lower off-white superfluous amorphous TiO_2_ NPs were collected separately and washed with ethanol and DI water, centrifuged and dried at 80°C. The annealed TZOs were also prepared by heating the as-prepared TZO at 300°C (TZO300) and 600°C (TZO600) for 2 hours in air to convert the amorphous TiO_2_ NPs to the anatase and rutile phase, respectively. The crystallization of as-prepared samples was characterized by X-ray diffraction (XRD, Philips, PW1813). Morphology and structure characterizations were carried out using a scanning electron microscope (SEM, Philips XL-30) and a high-resolution transmission electron microscope (HRTEM, JEOL 2010F) equipped with an energy-dispersive X-ray spectrometer (EDS). The photoluminescence (PL) was measured by a 325 nm He-Cd laser as the exciting light source. UV-Vis absorption spectrum measurement was conducted using a UV-Vis spectrophotometer (Lambda 20) and an integrating sphere (Labsphere) with a sampling spot of 10 mm × 10 mm at normal incidence. The surface area was characterized using a Brunauer-Emmett-Teller (BET) machine and a surface-pore size analyzer (Beckman Coulter, SA 3100). Chemical bonding information of the samples was obtained using a Fourier transform infrared spectrometer (FTIR; Bio-Rad, FTS 600). For the photocatalytic measurement, 80 mg of each catalyst was suspended in 200 mL of a methylene blue (MB) aqueous solution (20 ppm). The mixture was poured into a quartz tube and kept in a dark environment for 30 minutes to allow it to attain an equilibrium adsorption state. The concentration of the MB solution slightly decreased while it was in the dark room with the C_0_ value slightly lower than 20 ppm at t = 0. UV irradiation was carried out using a 500 W high-pressure Hg lamp (with the strongest emission at 368 nm) and cooling by cycling water. After a set irradiation time, ~3 mL of the mixture was withdrawn, and the catalysts were separated from the suspensions by a centrifuge. The degradation process was monitored by the UV-Vis spectrophotometer measuring the absorption of MB at 664 nm.

## Author Contributions

C.C. designed the research, performed TEM characterizations, and photocatalytic experiments, analysed the results and wrote the paper. A.A. and Z.X. performed the XRD and UV-Vis spectrophotometer characterization and the paper revision. C.Z. performed the nanomaterial fabrication. H.S. incorporated in the interpretation of experimental results. N.W. contributed significant discussion and final paper polishing.
